# Surface and chemical characteristics of platinum modified activated carbon electrodes and their electrochemical performance

**DOI:** 10.3906/kim-2103-14

**Published:** 2021-10-19

**Authors:** Tuğrul YUMAK, Serap YUMAK, Abdulkerim KARABULUT

**Affiliations:** 1 Department of Chemistry, Faculty of Arts and Science, Sinop University, Sinop Turkey; 2 Department of Interdisciplinary Nuclear Energy and Energy Systems, Institute of Graduate Studies, Sinop University, Sinop Turkey; 3 Department of Basic Sciences, Faculty of Science, Erzurum Technical University, Erzurum Turkey

**Keywords:** Activated carbon, packaged supercapacitors, Pt modified activated carbons, characteristics of metal-activated carbon composites

## Abstract

Platinum (Pt) loaded activated carbons (ACs) were synthesized by the thermal decomposition of platinum (II) acetylacetonate (Pt(acac)_2_) over chemically activated glucose-based biochar. The effect of Pt loading on surface area, pore characteristics, surface chemistry, chemical structure, and surface morphology were determined by various techniques. XPS studies proved the presence of metallic Pt^0^ on the AC surface. The graphitization degree of Pt loaded ACs were increased with the loaded Pt^0^ amount. The electrochemical performance of the Pt-loaded ACs (Pt@AC) was determined not only by the conventional three-electrode system but also by packaged supercapacitors in CR2032 casings. The capacitive performance of Pt@AC electrodes was investigated via cyclic voltammetry (CV), galvanostatic charge-discharge curves (GCD), and impedance spectroscopy (EIS). It was found that the Pt loading increased the specific capacitance from 51 F/g to 100 F/g. The ESR drop of the packaged cell decreased with the Pt loading due to the fast flow of charge through the conductive pathways. The results showed that the surface chemistry is more dominant than the surface area for determining the capacitive performance of Pt loaded AC-based packaged supercapacitors.

## 1. Introduction

Activated carbons (ACs) are most commonly used electrode material for electrochemical double layer capacitors (EDLCs) because they are commercially available, cheap and can be easily engineered [1]. However, they suffer from poor energy storage capacity and inferior rate capability [2]. Therefore, ACs, especially biomass-derived ACs are needed to be re-engineered to fabricate higher power density capacitors. EDLCs store the charge at the electrode/electrolyte interface through the reversible electrolyte ion adsorption on the pores of electrode [3]. It is concluded that both micro- and mesopores have been proven experimentally to provide efﬁcient ion transport for a high-power density while maintaining the high energy density [3,4]. Therefore, accessible surface area, total micro and meso pore volume, pore size, and distribution are key factors for high power and energy density. Improvement of the pore characteristics via chemical/physical activation, and synthesis of AC-based composite materials are the main approaches for producing re-engineered ACs.

It is well-known that only the electrochemically active surface area and micro-meso sized pores (<50 nm) are effective for electrochemical double layer formation [5] and ion transport. Heimböckel et al. have recently discussed the effect of pore size and electrochemically active surface area on the energy storage capacity of ACs in details [6]. It is concluded that the pores around 0.74 nm and 0.90 nm are the best for the highest capacitive performance, and the accessible surface area remains a key factor [6]. The direct activation of biomass can result in well-developed ACs that present surface areas up to 2500 m^2^/g, broad pore size distribution which consists of mostly micro and mesopores, and hetero-atom containing surface functional groups [7]. Numerous studies can be found focusing on the activation of biomass to produce high surface area ACs [8–13]. The dependence of the pore structure and surface area to the activation parameters is also discussed by Jin et al. [14]. However, no direct correlation between specific capacitance, and surface area, and pore size was found in previous studies. Therefore, changing our perspective by focusing on the modification of activated carbons will help to improve highly efficient supercapacitors. Instead of the improvement of the surface and the pore characteristics of ACs, the carbon-pseudocapacitive material composites (metal oxides/hydroxides/conducting polymers) are of great interest for the development of supercapacitors with enhanced energy storage capacity [15]. Several studies have reported the increased capacitive performance of composite materials composed of different types of carbon and pseudo-capacitive materials [16–19]. The composite materials not only overcome the problems of each component but also show better results due to the synergic effects [20].

Despite the high number of publications focusing on metal oxide-ACs composite electrodes only a limited number of works found related to AC-metal composites. Although it is reported that the AC/metal composite may exhibit high transfer characteristics and effective catalytic/adsorptive properties [21,22], it is thought that the difficulty of keeping the metal in the zero-valent state on AC surface may be a reason for the limited number of papers. Especially the oxygen-containing surface functional groups on the AC surface have great influence on the oxidation of transition metals [23]. Therefore, noble metals can be considered as a good choice because of their resistance to oxidation even higher temperatures. Among the noble metals, Pt is in great attention due to its catalytic activity on fuel cell applications [24,25], and H_2_ production [26,27]. However, only a few papers found which focused on the supercapacitor applications of Pt loaded carbon materials. Okajima et al. [28] reported that the CV curves of Pt loaded AC electrodes were mainly featureless between the hydrogen and oxygen evolutions which means no redox peaks were observed. Since the surface of the activated carbon includes electroactive oxygen-containing surface functional groups, it is thought that the pseudocapacitive contribution of these groups might be suppressed by the double-layer capacitance. Although it is reported that the loaded Pt particles were in 20 nm or less, but no information about surface chemistry was found [28]. Therefore, it is thought that to understand the increase in capacitive performance, the effect of Pt loading on the chemical and surface characteristics of AC should be displayed. Besides that, no other study focused on the packaged supercapacitor based on Pt modified AC electrodes was found. Investigating the electrochemical properties in packages systems will give more correct information about the usability of these electrodes in up-scale applications than the conventional three-electrode system. 

The aim of this work is to investigate the role of Pt loading on the change of surface and chemical properties of highly porous AC, and the electrochemical characteristics of packaged supercapacitor cells used the Pt loaded ACs as electrode materials. Glucose was used as carbon source instead of real biomass in order to eliminate the possible catalytic effect of minerals included in real biomasses [29]. The AC with high surface area was obtained by the chemical activation of glucose-derived pyrolytic carbon. Pt was loaded with different amounts onto the AC via the thermal degradation of platinum (II) acetylacetonate (Pt(acac)_2_). N_2_ physisorption, nonlocalized density functional theory (NLDFT) calculations from adsorption-desorption isotherms, Raman spectroscopy, X-ray photoelectron spectroscopy (XPS) techniques were used to characterize the synthesized metal-AC composites. The effect of Pt loading on the electrochemical properties was discussed over cyclic voltammetry (CV) and impedance spectroscopy (EIS) in three-electrode system, and the galvanostatic charge-discharge tests of packaged supercapacitors in CR2032 casings.

## 2. Materials and methods

### 2.1. Chemicals and reagents

D-(+)-Glucose (C_6_H_12_O_6_) (Sigma Aldrich, CAS Number: 50-99-7), potassium hydroxide (KOH) (Sigma Aldrich, CAS Number: 1310-58-3), hydrochloric acid (HCl) (Sigma Aldrich, CAS Number: 7647-01-0), platinum (II) acetylacetonate (Pt(acac)_2_) (Sigma Aldrich, CAS Number: 15170-57-7), carbon black (CB) (Alfa Aesar, CAS Number: 1333-86-4), poly(tetrafluoroethylene) (PTFE) (ACROS, CAS Number: 9002-84-0), N-methyl-2- pyrrolidone (NMP) (ACROS, CAS Number: 872-50-4) and Nafion NRE-212 membrane 0.05 mm thick (Alfa Aesar, CAS Number: 31175-20-9) were used as received without purification.

### 2.2. Preparation of glucose-based activated carbons

D-(+)-Glucose was subjected to pyrolysis at 500 °C for 60 min under the Argon environment at a 20 mL/min flow rate. The collected carbon powders after pyrolysis labelled as pyrolytic carbon (PC) was mixed with KOH solution for 6 h, the PC:KOH ratio is set to 1:4 (w/w). After 6 h mixing of the PC with KOH, the solution was dried at 105 °C for several hours in an oven. The dried PC+KOH mixture was subjected to thermal treatment at 800 °C under the Argon environment at a 50 mL/min flow rate. After cooling the reactor naturally to the room temperature collected powders were washed with hot distilled and 0.1 M HCl solution water until the pH reached 6.8–7.2. Then the powder was collected by filtering and dried at 105 °C in an oven overnight. The dried powders were labelled as GAC and stored in a sealed bottle.

### 2.3. Preparation of Pt loaded activated carbons

Pt was loaded onto GAC as described in somewhere else [30]. In this modified technique, proper amount of Pt(acac)_2_ in 20 mL ethanol was mixed with dispersed GAC in 20 mL distilled water for 12 h to complete the adsorption of Pt molecules. The stoichiometric weight ratio of loaded Pt to GAC was set as 1%, 3%, and 5%. The dried Pt loaded GAC samples were thermally treated at 300 °C for the formation of Pt^0^ species with the degradation of Pt(acac)_2_. Then the powders were collected and labelled as GAC1, GAC3, and GAC5, respectively. In order to discuss the effect of thermal treatment on the chemical, surface, and electrochemical properties of GAC sample was subjected to thermal treatment at the same conditions without Pt loading. The sample was labelled as GAC-t.

### 2.4. Fabrication of supercapacitor in CR2032 casings

The electrodes were prepared by a thin film casting method as described in a previous study [23]. The as-synthesized AC samples (GAC, GAC-t, GAC1, GAC3, and GAC5) were mixed with CB and PTFE in a ratio of 85:5:10 (w/w) in NMP to form a homogeneous electrode slurry. The total mass of the electrode slurry and activated carbon in the slurry are 1 g and 0.85 g, respectively. The mixture was mixed for 24 h on a magnetic stirrer and cast on SS304 plate with 0.70 mm thickness. The film was dried in air for 2 days and further in an oven overnight. The electrodes (13 mm in diameter) (dry mass is almost 0.0020 g) and Nafion membrane (16 mm in diameter) were punched and soaked in 6 M KOH electrolyte for 24 h. Finally, the supercapacitors were assembled in CR2032 casing and sealed with a hydraulic press. A general schematic presentation of the fabrication process of a supercapacitor in CR2032 can be found somewhere else [31].

### 2.5. Characterization

The BET surface area and pore characteristics were evaluated with a Micromeritics 3Flex volumetric gas adsorption analyser based on HK83 points method through N_2_ physisorption at 77 K. Prior to the measurements, all samples were degassed at 105 °C for 24 h. Adsorption-desorption isotherms were used in NLDFT calculations to determine the pore volume distribution. X-ray photoelectron spectroscopy (Specs-Flex XPS using Al Kα (1486.6 eV) radiation, survey scan with a 1 eV step and detailed scan with a 0.1 eV), Raman (WITech alpha 300R with 532 nm Ar laser excitation) techniques were applied in order to investigate the effect of Pt loading and experimental conditions on the surface chemistry, chemical structure and surface morphology. The electrochemical testing of the fabricated supercapacitors was conducted on the Gamry Reference 1010(E) as outlined in supercapacitor testing standard IEC 62391-1:2006. Cyclic voltammetry and impedance spectroscopy techniques were used for determining the electrochemical properties. The electrochemical performance was evaluated by constant current charge-discharge test for 2500 cycles of a 0.1–1.0 V working voltage window at a 100 mA/g current density. The gravimetric specific capacitance (C_g_/Fg^–1^) was calculated from Eq. (1) after excluding the ohmic part, equivalent series resistance (ESR).

(1)Cg=2l(dV/dt)m

where C_g_ represents the gravimetric specific capacitance, I is the discharge current, m is the average mass of one dry electrode, and dV/dt is the rate of voltage change.

## 3. Results and discussion

### 3.1. Surface area and pore characteristics 

The BET surface area and pore volume characteristics of synthesized activated carbons are shown in Table 1. As seen in the table, the thermal treatment caused ~20% increase in the BET surface area (GAC and GAC-t). In addition, a significant increase in the t-plot micropore area, total pore volume and micropore volume was observed. This may be attributed to the formation of CO_2_ due to the degradation of carboxylic acid, lactone, and anhydride species with the increasing temperature on the AC surface [32,33]. As a consequence, the evolved CO_2_ gas may lead to the creation of new micropores. The decrease in the average pore diameter may be related to the partly re-arrangement or shrinkage of pores. However, it is thought that the statistical analysis of experimental data is needed to regard the change in pore diameter is meaningful. It is obvious that Pt loading caused a slight decrease in surface area for the GAC1 sample and a dramatic decrease for GAC3 and GAC5 samples. The same trend was observed in other parameters such as t-plot micropore area, total pore volume, micropore volume, and pore diameter. Therefore, it is thought that the loaded Pt particles were positioned in the internal part of the pores [34]. 

**Table 1:** Surface area and pore volume characteristics of synthesized activated carbons.

Sample	BET surface area (m2/g)	t-Plot micropore area (m2/g)	Total pore volume (cm3/g)	Micropore volume (cm3/g)	Pore diameter (4V/A) (nm)
GAC	629.11	504.80	0.28	0.22	1.86
GAC-t	756.80	637.56	0.35	0.28	1.85
GAC1	745.17	629.59	0.35	0.27	1.85
GAC3	634.08	536.33	0.29	0.23	1.85
GAC5	631.50	526.03	0.29	0.23	1.84

**Figure 1:**
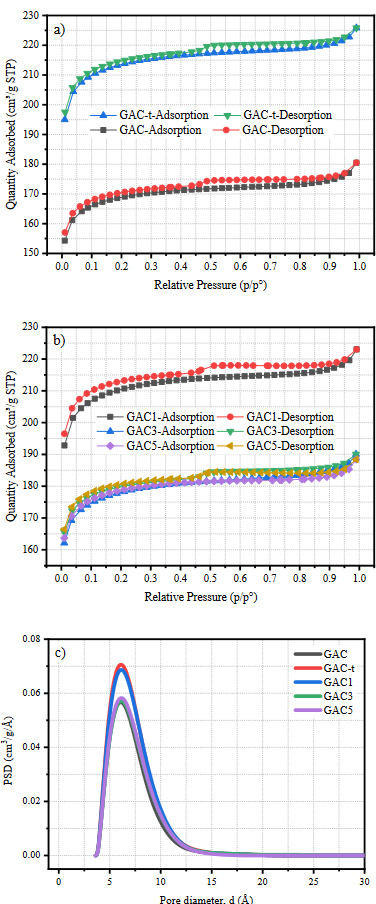
Adsorption-desorption isotherms of a) untreated and thermally treated activated carbon, b) Pt loaded activated carbons, and c) pore volume distributions calculated from NLDFT.

Figure 1 shows the adsorption-desorption isotherms, and the pore volume distributions calculated from the NLDFT studies of all samples. According to the IUPAC classification, all samples display a Type-IV isotherm which is typical for activated carbons, and turns into a Type-II isotherm at high p/p^0^ as a result of monolayer-multilayer adsorption on mesopore walls [35,36]. A clear decrease step was observed between 0.4–0.6 p/p^0^ relative pressures corresponds to the uniform mesoporous structure [37]. In addition, the H4 loops which are typical for micro-mesoporous activated carbons [35] were observed in all samples. As seen in Figure 1a, the increase of adsorbed gas amount verifies the increase in surface area with the thermal treatment. In opposite, the decrease in the adsorbed gas amount with the Pt loading can be seen in Figure 1b. Almost the same shape of the isotherms reveals that the Pt loading did not affect the pore characteristics of activated carbon samples. In Figure 1c, it is clear that all samples are consist of mostly micropores. In addition, it seems that the thermal treatment and the Pt loading affected the distribution of micro-sized pores. Since the Pt loading caused a decrease only in the micropore region, it can be said that loaded Pt particles may be maximum 12.5 nm in size. The calculated NLDFT results are compatible with Table 1 (GAC-t, GAC1, GAC3, and GAC5), and previously published works [28].

### 3.2. Surface chemistry 

**Figure 2:**
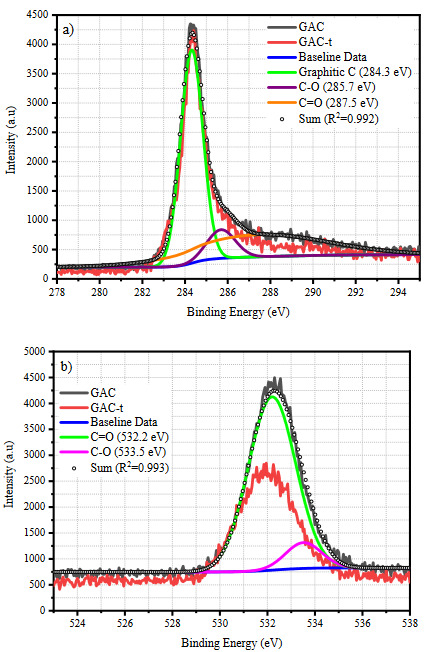
a) C1s and b) O1s spectra of GAC and GAC-t samples. The deconvolution peaks of C1s and O1s spectra are belong to GAC sample.

The effect of Pt loading on the surface chemistry of AC, the surface functional groups (SFGs), the amount and the chemical state of loaded Pt was determined by XPS. The C1s and O1s spectra of GAC and GAC-t samples were represented in Figure 2. Also, the deconvolution of C1s and O1s spectra for the GAC sample can be seen in Figure 2. Three main peaks indicating graphitic carbon at 284.3 eV, C-O- groups at 285.7 eV, and C=O groups at 287.5 eV were found in the C1s spectra of GAC sample (Figure 2a) [38]. It can be said that there is no significant effect of thermal treatment on the C1s spectra was observed. A slight change at ~287–288 eV was detected as a result of a change in the relative amount of C = O groups. The relative amounts of SFGs will be discussed in detail later. Figure 2b shows the O1s spectra of GAC and GAC-t, and the deconvolution results for GAC sample. Two main peaks corresponding to the C = O at 532.2 eV, and C–O at 533.5 eV [39] were observed. It is evident that the shape of the O1s peak changed significantly with thermal treatment. This change may be attributed to the degradation of carboxylic acid species with thermal treatment.

**Figure 3:**
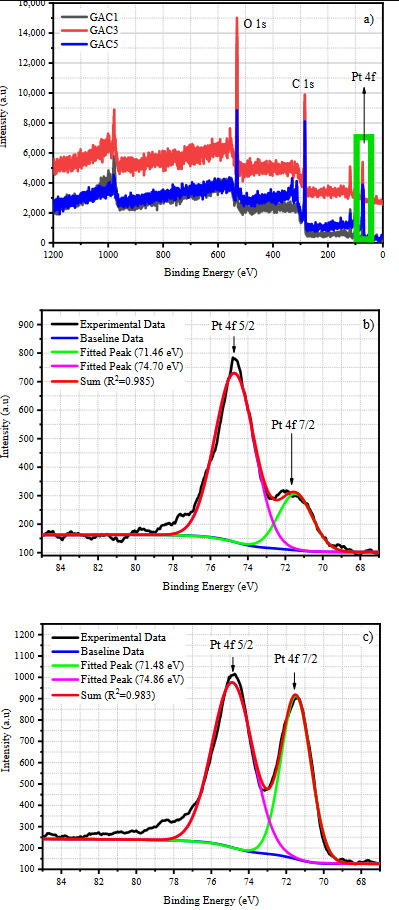
a) XPS survey spectrum of Pt loaded samples, and the deconvolution of Pt 4f spectra of b) GAC3 and c) GAC5 samples.

The survey spectra of Pt loaded samples and the deconvolution of Pt 4f spectra of the samples, GAC3 and GAC5 can be seen in Figure 3. As seen in the figure, the Pt 4f peak was observed for all Pt loaded samples at ~75 eV which proves the successful loading of Pt on activated carbons. The intensity of Pt 4f peaks increased with the increasing Pt amount in the sample. Two characteristic peaks located at 71.46 eV (in Figure 3a) and 74.70 eV (in Figure 3b) for GAC3, and 71.48 eV and 74.86 eV (in Figure 3c) for the GAC5 sample corresponds to the presence of Pt^0^ formation [30]. 

Table 2 shows the results of peak deconvolution, the relative amount of surface functional groups and the atomic amounts of C, O, and Pt. As mentioned in Figure 2, the relative amount of C=O groups was 35.1% in GAC and decreased to 5.5% in the GAC-t sample. Accordingly, the increase in the relative amounts of graphitic C (C=C) and C-O- groups was observed. Among the SFGs, although the carbonyl groups are electrochemically inactive, they increase the wettability of electrode [40] and help to enhance the capacitive performance indirectly. The hydroxyl groups in hydroquinone, phenol, and alcohol species assist to increase the specific capacitance by creating pseudo-capacitance via redox reactions [41]. Moreover, it is thought that the increasing relative amount of graphitic carbon increased the resonance states of electrons and the circulation of electrons, therefore resulted in an increase in the capacitive performance. The relative amount of loaded Pt^0^ for GAC1, GAC3, and GAC5 were found 0.54%, 1.14%, and 2.88%, respectively, from the survey scan. These results prove that the selected method is to succeed in loading Pt^0^ form onto activated carbon. It can be seen from the table that the relative amount of SFGs changed with the loaded Pt amount. The change in surface chemistry can be mainly explained by the catalytic effect of Pt on the thermal degradation of activated carbon [27]. On the other hand, thermal treatment, itself, affects the surface chemistry, thus caused a change in the SFGs [23]. An increase in the relative amount of carbon and a decrease in the oxygen was observed with the thermal treatment. This may be attributed to the release of H_2_O trapped between the porous structure and then the release of CO and CO_2_ [32]. Ruiz et al. [32] and Figueiredo et al. [33] have reported the release of CO and CO_2_ via the degradation of carboxylic acid, lactone, and anhydrides at 300 °C during the thermal treatment of activated carbons. This result shows good consistency with our XPS results.

**Table 2:** Deconvolution results of C1s spectra of all samples.

Sample	Atomic amount (%)			C=C (Graphitic carbon)			C-O-(Phenol – Alcohol – Ether)			C = O		
	C	O	Pt0	BE (eV)	fwhm	Area (%)	BE (eV)	fwhm	Area (%)	BE (eV)	fwhm	Area (%)
GAC	61.53	38.47	-	284.3	1.24	55.5	285.7	1.55	9.4	287.5	7.56	35.1
GAC-t	74.30	25.70	-	284.4	1.26	82.7	285.9	1.14	11.8	287.2	1.38	5.5
GAC1	63.68	35.78	0.54	284.4	1.34	74.2	286.0	1.46	14.8	288.0	2.91	11.0
GAC3	65.75	33.11	1.14	284.7	1.99	67.3	286.3	6.58	32.7	-	-	-
GAC5	69.35	27.77	2.88	284.6	1.85	80.2	285.7	2.44	19.8	-	-	-

### 3.3. Chemical structure 

**Figure 4:**
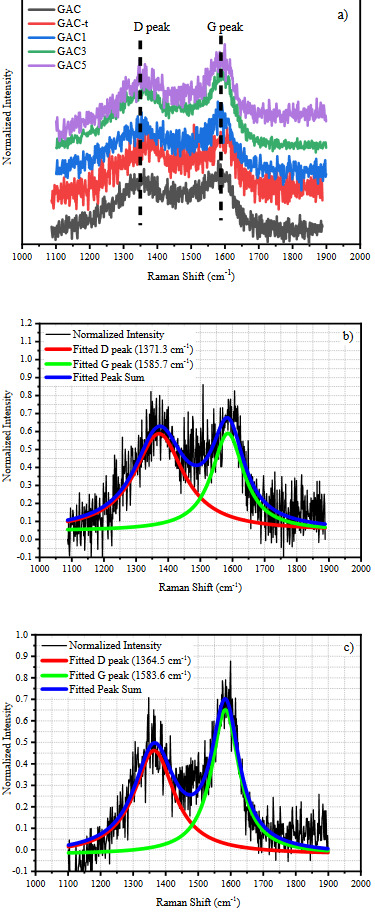
a) Raman spectrum of all samples (1100–1900 cm–1), example deconvolution of b) GAC-t and, c) GAC5 samples.

The Raman spectra of all samples and example deconvolutions of GAC-t and GAC5 samples were presented in Figure 4. As seen in Figure 4a, the D (disorder) band resulting from the defects in sp^2^ structure at around 1360 cm^–1^ and G (graphitic) band caused by vibrations in the plane of graphite plates at around 1580 cm^–1^ were observed [42]. The overlapped D and G bands without a clear separation are typical for heterogeneous carbon microstructure and disordered activated carbons [43]. Examination of the change in signal intensity of the D band (relative to the G band) allows the relative measurement of defects in the graphitic material. Therefore, all Raman spectra were deconvoluted into two Lorentzian in order to determine the I_D_/I_G_ to express the change in chemical structure quantitative. Figures 4b and 4c shows the example deconvolutions for GAC-t and GAC5 samples. Table 3 shows the peak positions, normalized peak intensities and the calculated I_D_/I_G_ ratios for all samples.

**Table 3:** Deconvolution results of C1s spectra of all samples.

Sample	D band		G band		ID/IG
	ν/cm–1	NPI *	ν/cm–1	NPI *	
GAC	1359.9	0.46	1576.6	0.47	0.98
GAC-t	1371.3	0.59	1585.7	0.59	1
GAC1	1360.5	0.49	1586.3	0.55	0.89
GAC3	1360.2	0.59	1586.3	0.68	0.87
GAC5	1364.5	0.46	1583.6	0.65	0.71

Although a significant shift in the peak positions of D and G band with the thermal treatment were observed, the I_D_/I_G_ ratio remained nearly the same indicating that thermal treatment has no significant effect on the chemical structure of glucose-based activated carbons. A correlation can be seen between the I_D_/I_G_ ratios and peak positions of Pt loaded AC samples. The increasing Pt amount led to a decrease in the I_D_/I_G_ ratio, this indicates the increase of highly ordered graphitic planes in AC structure. It is clear that the Pt loading has a catalytic and synergetic effect on the change in chemical structure.

### 3.4. Electrochemical tests

**Figure 5:**
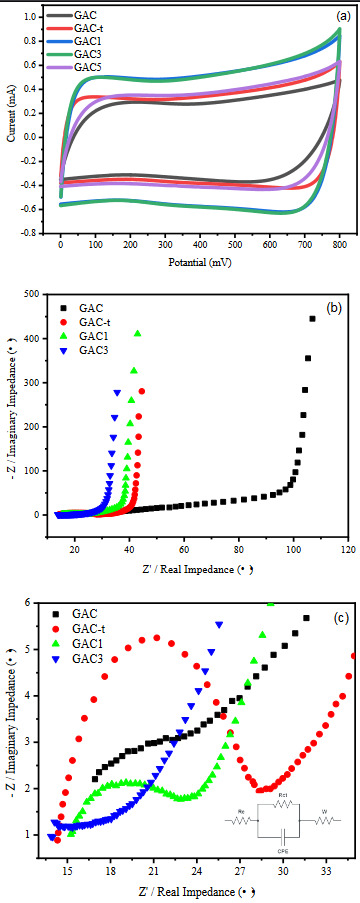
Electrochemical performance of the as-synthesized samples in a three-electrode system: (a) CV curves at 10 mV/s; (b) Nyquist plots from impedance spectroscopy (EIS) data; (c) The high frequency region of (b)

The electrochemical performance of the samples in three-electrode system can be seen in Figure 5. Figure 5a shows the CV curves in the potential window of 0 V to 0.8 V with a scan rate of 10 mV/s. The rectangular shape of the curves indicates a good capacitor behaviour and the double-layer capacitance behaviour. However, as mentioned above, voltage humps at between 200–600 mV were observed due to the presence of oxygen-containing functional groups which proved with XPS results. This result match very well with the previous studies [44,45]. In addition, it is obvious that the area under the CV curves getting larger with thermal treatment (GAC-t, red line) and Pt loading (GAC1, blue line; GAC3, green line; GAC5, purple line). It can be easily seen that GAC1 and GAC3 sample exhibits higher specific capacitance compared to others. This may be attributed to the change in surface chemistry and porous characteristics. The Nyquist plots electrochemical impedance spectroscopy (EIS) data were used to the determine the combined resistance (R_s_) of intrinsic resistance of electrode materials, ionic resistance of electrolyte and contact resistance between the electrode and current collector, and the electrode conductivity and charge-transfer resistance (R_ct_) [46]. It can be seen from the low frequency region (Figure 5b) of the Nyquist plots that all samples showed ideal capacitor behaviour. At the intermediate frequency region, the Warburg line (45 ° slope) and following nearly straight vertical line were observed for GAC-t, GAC1, and GAC3 indicating that all samples possess ideal capacitor behaviour and allow the solution ions to be adsorbed by the electrode [47]. A long Warburg line originated from the lower electric conductivity was observed for GAC sample [47]. The Warburg line shortened with the Pt loading and this proves the correlation between the electric conductivity and loaded Pt amount. In Figure 5c, it is obvious that the diameter of the semicircle decreased at high frequency region with the Pt loading implying that the Pt modified electrodes have lowest internal resistance and highest electric conductivity.

**Figure 6:**
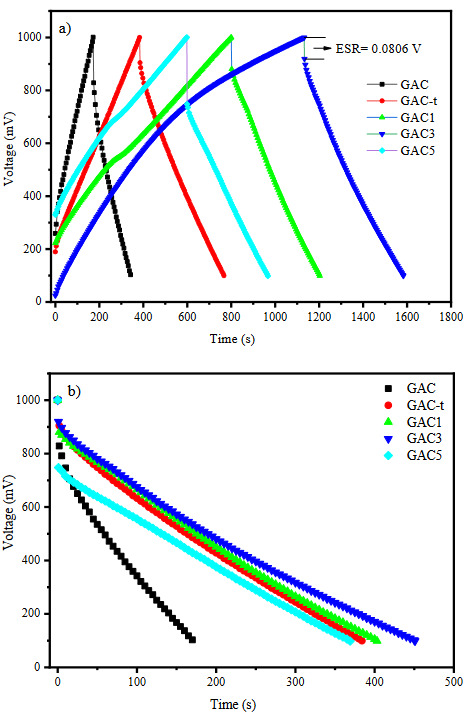
a) Charge-discharge, b) discharge curves of all packaged supercapacitors with synthesized activated carbons

Figure 6 displays the constant current charge-discharge profiles of packaged supercapacitors using GAC, GAC-t, GAC1, GAC3, and GAC5 samples as symmetric electrodes. Both charge and discharge curves can be seen in Figure 6a, while only the discharge profiles were presented in Figure 6b in order to better compare the capacitive performance. All the curves belong to the 501st cycle of the 2500 cycle test regime. The charge-discharge curves showed a repeatable cyclic behaviour and the characteristic symmetrical triangular shape indicating a high rate capacitive performance [48]. However, some minor distortions in the charge curves were observed. These distortions are also a proof for the decrease in the coulombic efficiency. The calculated coulombic efficiency for the GAC, GAC-t, GAC1, GAC3, and GAC5 samples are 98.8%, 99.6%, 79.8%, 65.1%, and 71.7%, respectively. It is obvious that the hydrothermal treatment caused an increase and Pt loading caused a decrease in the Coulombic efficiencies. These can be attributed to the different adsorption/desorption rates of electrolyte on the electrode surface, the surface chemistry of the electrode [49], and the conductive pathways occurred by Pt loading. Although the Pt loading caused a decrease in the specific surface area, a significant change in the pore structure and mean pore diameter was not observed. Therefore, it is thought that the capacitive performance is mostly affected by the synergistic effect of surface chemistry and Pt loading.

The discharge time, which is given in Figure 6b gives a hint to estimate the specific capacitances of supercapacitors. It is seen that the GAC3 based supercapacitor has the highest discharge time. The specific capacitances of all samples were calculated by using Eq. (1) given. The specific capacitances were calculated as 51 F/g, 100 F/g, 105 F/g, 118 F/g, and 98 F/g for GAC, GAC-t, GAC1, GAC3, and GAC5 based supercapacitors, respectively. It is obvious that the Pt loading led to an increase in the capacitive performance of supercapacitors. However, a dramatic decrease was observed for the GAC5-based supercapacitor. This may be attributed to the higher equivalent series resistance (ESR) drop which is related to the electrolyte resistance, the collector/ electrode contact resistance, the electrode/electrolyte interface resistance, surface functional groups [50], electrical conductivity, and pore characteristics [51]. The ESR drop for GAC, GAC-t, GAC1, GAC3, and GAC5 based supercapacitors were found as 0.1724 V, 0.0955 V, 0.1197 V, 0.0806 V, and 0.2527 V, respectively. The increase in the ESR drop with the increasing Pt amount may be attributed to the increasing conductive paths on activated carbon surface and channels. It is thought that the stored charge can easily flow through these pathways resulting in higher ESR drop.

The energy and power densities were calculated by using the formulas given somewhere else [52]. The energy densities are 5.74, 11.25, 11.81, 13.28, and 11.03 Wh kg^−1^ and the power densities are 98.36, 105.47, 105.52, 105.96, and 107.56 W kg^−1^ for the samples GAC, GAC-t, GAC1, GAC3, and GAC5, respectively. It can be clearly seen that the energy and power densities are increased with the Pt loading.

Since the electrochemical performance of an activated carbon-based supercapacitor is rely on the surface area, pore characteristics, and surface chemistry, it is very important the find out the effect of the loaded materials on these properties. In this work, the Pt loading caused a decrease in the surface area but no significant effect on the pore characteristics. It is also obvious that the Pt loading has changed the relative amount of surface functional groups of activated carbon. These results are in good consistent with previously reported works [25]. It is thought that the increase in the capacitive performance can be attributed to the formation of hydrophilic Pt-OH groups due to the Eq. (2) [53]. The hydrophilic groups led to a higher adsorption rate of electrolyte on the surface, and the increase in the specific capacitance as a consequence. However, with the increasing Pt amount, the reaction between the Pt-OH and CO groups (Eq. (3)) caused the formation of CO_2_ and Pt active centres [53]. The increasing amount of Pt active centres may increase the pathways of charge transfer from electrodes to the current collectors, thus caused higher ESR drops.

*Pt *+ *H* *_2_* *O → Pt *- *OH*+*H* ^+^+*e* ^-^⋅ (2)

*Pt* - *OH *+ *Pt *- *CO *→ *2Pt* + *CO* *_2_* + *H* ^+^ + *e* ^-^⋅ (3)

**Figure 7:**
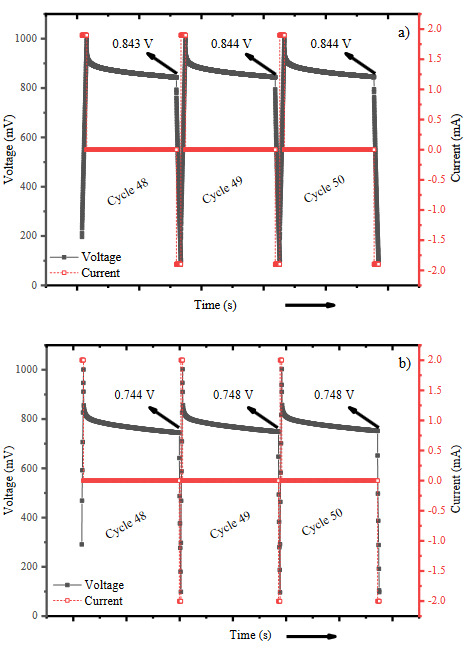
Self-discharge (voltage holding) tests for a) GAC-t, b) GAC3 sample-based supercapacitors.

The self-discharge behaviour or in other words voltage holding character defines the capability of sustaining a constant charge hold, and is a significant factor for determining the performance evaluation. The fabricated supercapacitors were charged to 1V at a constant current density of 100 mA/g. The voltage loss against to the time was recorded during 150 min hold time. Figure 7 shows the 48th, 49th*,* and 50th self-discharge curves of 50 cycles regime for GAC-t and GAC3 sample. The voltage loss characteristics did not change more than 4% over the 50 cycles indicating that the electrochemical performance of the supercapacitors was highly stable. As can be seen from the figure, Pt loading caused a 11% decrease in the voltage holding which may be attributed to the increasing electrical conductivity with the Pt loading. This result is also in good correlation with the EIS results.

## 4. Conclusions

In this work, the Pt loaded activated carbon materials were synthesized with different compositions and characterized by N_2_ physisorption, XPS, and Raman spectroscopy techniques. The fabricated supercapacitors were subjected to constant charge-discharge tests in order to determine electrochemical performance. It is found that the Pt loading method (thermal treatment) has a positive effect on the surface characteristics and electrochemical performance as expected. The Pt loading method, itself, has changed the surface chemistry by the decomposition of surface functional groups. It is found that the Pt loading led to a 130% increase in capacitive performance. The Pt loading significantly changed the surface chemistry. The reactions seen in Eq. (2) and (3), are both responsible for the change in surface chemistry, increase in the capacitive performance. It is evident that the loaded Pt amount is the decisive factor for determining the electrochemical performance of fabricated supercapacitors. In addition, is thought that the Pt loading method is highly effective on the surface chemistry of activated carbon and chemical state of Pt.
